# Enhanced recovery in patients with gestational diabetes mellitus and *MTHFR* 677 TT genotype after taking high-dose folic acid supplements during mid-late pregnancy: an open-label interventional study

**DOI:** 10.3389/fendo.2023.1007192

**Published:** 2023-09-25

**Authors:** Jun Ying, Jie Zhang, Piyu Li, Lu Liu, Yan Li, Winnie W.Y. Lau, Qiao Chu, Benqing Wu, Xiaonan Wang, Hui Zhang

**Affiliations:** ^1^ School of Public Health, Shanghai Jiao Tong University School of Medicine, Shanghai, China; ^2^ Faculty of Medical Laboratory Science, College of Health Science and Technology, Shanghai Jiao Tong University School of Medicine, Shanghai, China; ^3^ Institute of Biological and Medical Engineering, Guangdong Academy of Science, Guangzhou, China; ^4^ Department of Obstetrics, University of Chinese Academy of Sciences Shenzhen Hospital, Shenzhen, China; ^5^ Cambridge Stem Cell Institute, Jeffery Cheah Biomedical Centre, Cambridge, United Kingdom; ^6^ Institute of Translational Medicine, University of Chinese Academy of Sciences Shenzhen Hospital, Shenzhen, China

**Keywords:** gestational diabetes mellitus, folic acid supplement, methylenetetrahydrofolate reductase (MTHFR) gene, recovery, diet

## Abstract

**Objective:**

To explore the relationship between folic acid supplementation and the recovery rate of gestational diabetes mellitus (GDM) in women with methylenetetrahydrofolate (*MTHFR*) 677 TT genotypes in mid-late pregnancy.

**Methods:**

9, 096 pregnant women were recruited with their *MTHFR* gene genotyped. 5,111 women underwent a 75-g oral glucose tolerance test (OGTT) and 2,097 were confirmed with GDM. The association between *MTHFR* genotypes and GDM risk was estimated using logistic and log-binomial regression, with age and parity set as the covariates to control their confounding effects. Further assessment of GDM risk on glucose levels was done using the ANCOVA model. As an open-label intervention study, 53 GDM patients with TT genotype were prescribed 800μg/day of folic acid as the high-dose group, while 201 GDM patients were given 400μg/day as the standard-dose group at their 24-28 weeks of pregnancy. A rate ratio (RR) of GDM recovery was estimated at each available time point for both groups. The time-to-GDM persistence events were analyzed with the Kaplan-Meier method and Cox-regression model. The trend of glucose levels over time was estimated using the linear model.

**Results:**

*MTHFR* 677 TT genotype has no significant association with the glucose levels and GDM risk, with an adjusted OR of 1.105 (95% CI 0.853, 1.431; p=0.452) and an adjusted PR of 1.050 (95% CI 0.906, 1.216; p=0.518) compared to the wildtype CC group. Patients in the high-dose group (n=38; 15 drop-outs; 40.69 days (95% CI 33.22, 48.15)) recovered from GDM approximately 27 days faster than those in the standard-dose group (n=133; 68 drop-outs; 68.09 days (95% CI 63.08, 73.11)). Concomitantly, the RR of GDM recovery rose and reached 1.247 (95% CI 1.026, 1.515) at 100 days of treatment with the standard-dose group as reference.

**Conclusion:**

High-dose folic acid supplement intake in mid-late pregnancy is associated with faster GDM relief in patients with *MTHFR* 677 TT genotype compared to the standard dose, which would be served as a novel and low-cost alternative therapy for the treatment of GDM.

## 1 Introduction

Gestational diabetes mellitus (GDM) is a metabolic disorder where hyperglycemia develops during pregnancy. It affects more than 10% of pregnancies worldwide (1) and is more common in southern China, with a prevalence of 13.7% in Shenzhen and 38.4% in Guangzhou under the latest IADPSG criteria (2). In addition to a higher risk of adverse perinatal outcomes, the offspring are more likely to develop diabetic embryopathy, especially anencephaly, microcephaly and congenital heart disease (3). Although healthy diets and physical exercises help maintain blood glucose levels, insulin therapy would be required if the blood glucose levels remain high (4). However, insulin therapy could be costly, may lead to side effects, such as weight gain or hypoglycemia, and is unsuitable for people with trypanophobia. Hence, novel therapeutic strategies for the treatment of GDM are urgently in need to improve the health conditions of both pregnant women and their offspring.

Folate, also known as vitamin B-9, is required for the maintenance of S-adenosyl-L-methionine (AdoMet)-dependent methylation and is enriched in a variety of fruits and vegetables (5). Its synthetic form, folic acid, is widely used as a dietary supplement (6). Inadequate blood levels of folate lead to a higher risk of developing neural tube defects (NTDs) during early pregnancy. Thus, folic acid supplements in addition to folate from the diet are required to prevent NTDs in the standard preconception and obstetrical care. However, there are no standard dosing instructions for the use of folic acid supplements. Daily supplementation with 400μg folic acid is mostly recommended 2-3 months before conception and throughout pregnancy (7–10), and pre-pregnancy intake of folic acid has been found to be associated with GDM risk to a high degree (11). Although the preventive role of folic acid to NTDs is well-studied, whether folic acid supplementation in early pregnancy (1~13 weeks) could reduce the GDM risk during the second/third trimester of pregnancy is still controversial (6, 12, 13), and the dose or duration-related association between folic acid supplementation and GDM risk remains unclear (14).

Methylenetetrahydrofolate reductase (*MTHFR*) is a key enzyme in folate metabolism, which catalyzes methylenetetrahydrofolate to methyltetrahydrofolate – a metabolically active form of folic acid, in the homocysteine methylation reaction. The *MTHFR* gene contains two main polymorphisms attributed to single nucleotide substitutions: 1) at the 677 position with cytosine substituted by thymine (C677T) and 2) at the 1298 position with alanine substituted by cytosine (A1298C). C677T, the most common variant in the *MTHFR* gene, occurs in approximately 8% of the population, and is known to reduce the catalytic activity of the enzyme (15). Since there are two copies of the *MTHFR* gene, *MTHFR* 677 has three genotypes: *MTHFR* 677 CC, *MTHFR* 677 CT and *MTHFR* 677 TT. It has been reported that T alleles of *MTHFR* 677 tend to increase susceptibility to GDM (16, 17). However, little is known if folic acid intake contributes to lower GDM risk in pregnant women with *MTHFR* C677T polymorphisms and moreover, if a pregnant woman with *MTHFR* 677 TT genotype was diagnosed with GDM, would higher folic acid intake be effective in terms of GDM relief, which was defined by negative OGTT follow-up test results.

To address the questions above, we designed an open-label interventional study offering 800μg/day of folic acid supplements to GDM patients with the *MTHFR* 677 TT genotype. A standard 400μg/day folic acid supplements was used as a control. We found that high-dose folic acid supplement intake (800μg/day) in the second-third trimester during pregnancy is associated with better GDM relief in patients with GDM and the *MTHFR* 677 TT genotype compared to patients with the same genotype given the standard dose of folic acid (400μg/day). This finding can provide guidance to future clinical trials, that high-dose folic acid supplementation in mid-late pregnancy could offer pregnant women with *MTHFR* 677 TT genotype a novel and low-cost approach for the treatment of GDM.

## 2 Materials and methods

### 2.1 Participant recruitment

**Figure 1:**
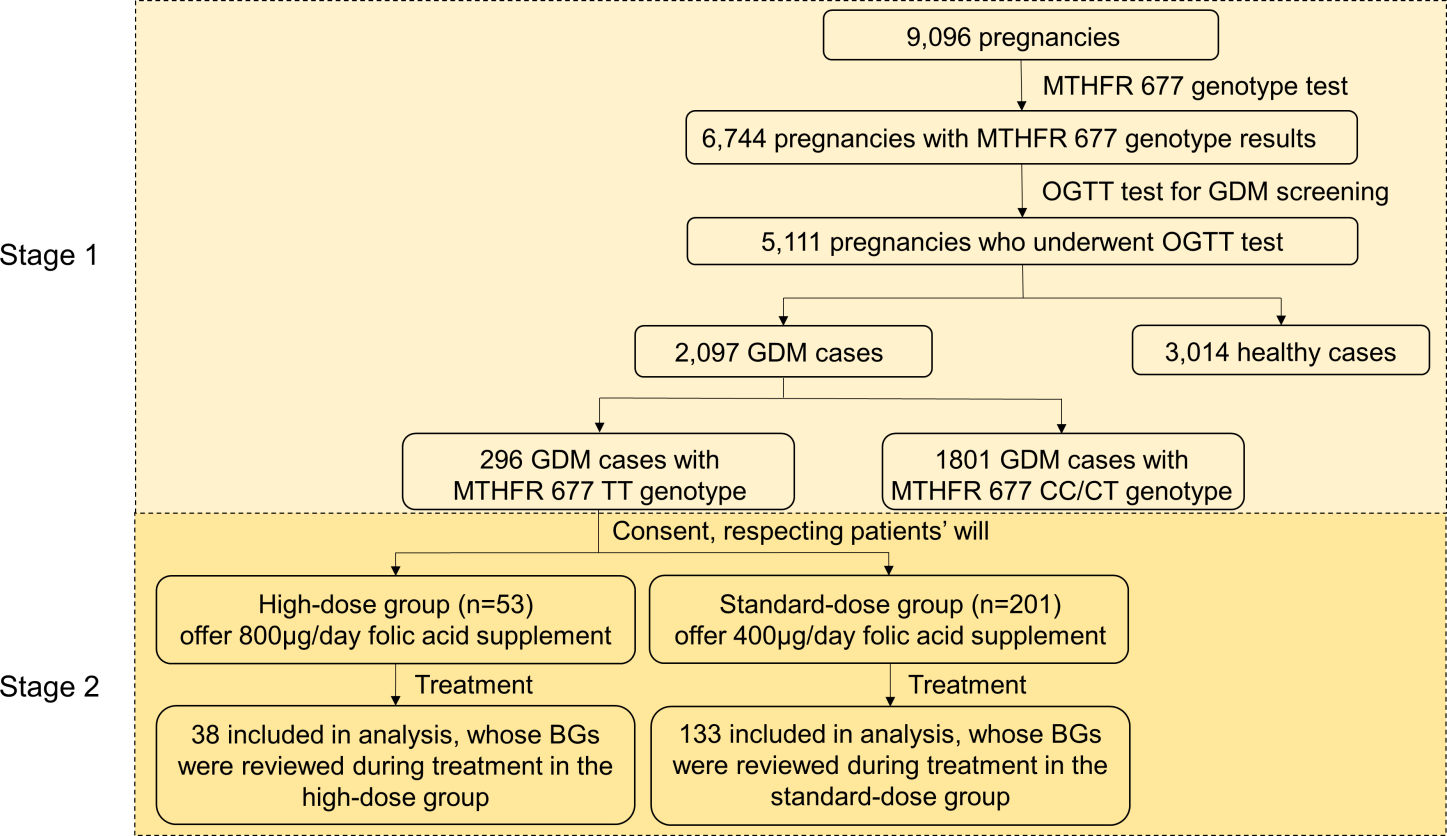
Study design. GDM patient selection based on inclusion/exclusion criteria described under the Materials and Methods. Stage 1: a retrospective cohort study; Stage 2: a prospective case-control interventional study. BG, blood glucose.

This study was conducted with the approval of the Ethics Committee of the University of Chinese Academy of Sciences, Shenzhen Hospital, with serial number LL-KT-2022036. 9,096 pregnant women were recruited from the University of Chinese Academy of Sciences-Shenzhen Hospital from 2017 to 2019 (**Figure 1**) and underwent *MTHFR* C677T mutation assessment. The *MTHFR* 677 polymorphism was assessed using the fluorescence quantitative polymerase chain reaction (FQ-PCR) toolkit (Kuang Yuan Diagnostics). Based on the test results, these pregnant women were classified into three groups (TT, CT and CC) based on their *MTHFR* 677 genotypes. At 24 weeks of pregnancy, the fasting blood glucose (FBG) and postprandial blood glucose (PBG) of 5,111 pregnant women were examined. GDM was diagnosed using a 75-g oral glucose tolerance test (OGTT), defined as FBG > 5.1mmol/l or 1 hour PBG > 10mmol/l or 2 hours PBG > 8.5mmol/l (18). In total, 2,097 pregnant women were confirmed with GDM.

### 2.2 Trial design

An open-label interventional study with a controlled selection of participants was designed to further assess the effectiveness of a higher dosage of folic acid intake in terms of GDM relief for GDM patients with *MTHFR* TT genotype in the late second trimester and the third trimester. 254 patients with TT genotype at 24-28 weeks of pregnancy agreed to participate from 296 pregnancy women with the *MTHFR* TT genotype. With written informed consent in accordance with the guideline, 53 patients were selected as the high-dose group and given 800μg/day folic acid supplement, whereas 201 patients were set as the standard-dose group and given 400μg/day folic acid supplement until delivery. Due to ethical restrictions, the intervention was not randomly allocated or blinded. Blood, liver and kidney function tests were suggested to the participants when recruited and at 31 and 37 weeks of pregnancy. 38 patients from the high-dose group and 133 from the standard-dose group repeated OGTT at least once and were used for downstream analysis.

### 2.3 Inclusion/exclusion criteria

Participants were only selected if they matched the following criteria: 1) diagnosed as GDM at 24-28 weeks of pregnancy; 2) no previous diabetes diagnosis, with their glycosylated hemoglobin (HbA1c)<6.5% (n=98) within 30 days before or after GDM screening; 3) has the *MTHFR* C677 TT phenotype; 4) aged 18-45 years; 5) didn’t have other chronic diseases or complications except for hypocalcemia; 6) were not on any additional medications apart from multi-vitamins that contains the prescribed amount of folic acid supplement; 7) no drinking or smoking history; 8) willingness to participate in the trial and comply with the folic acid intake requirements as suggested in the program. In addition to the baseline OGTT records, participants were required to have at least one extra OGTT result at a later stage with either FBG, 1-hour PBG or 2 hours PBG recorded.

### 2.4 Trial outcome

The primary endpoint of the trial was if participants were either no longer diagnosed with GDM or had reached the end of pregnancy (we have set this to be 100 days after recruitment at 24-28 weeks of pregnancy). Consistent with the initial diagnostic standard, patients with their abnormal glucose levels back to normal ranges (FBG<= 5.1mmol/L or 1-hour PBG<= 10mmol/L or 2hours PBG<= 8.5mmol/L) were considered as relieved from GDM.

### 2.5 Statistical analysis

#### 2.5.1 Observational association analysis

5,111 pregnant women with the *MTHFR* 677 genotype were included in the general observation study. The prevalence of each genotype group was calculated as a contingency table using the crosstab function in SPSS (v22). The odds ratios (OR) were estimate by logistic regression models to investigate the association between the *MTHFR* genotypes and GDM risk using the SPSS (v22). In the null logistic regression model, the *MTHFR* genotype was used as the univariate, while in the alternative model, age and parity were considered as covariates. The maternal age was sub-divided into three categories:<30, 30-34 and >34, while parity was set as a binary factor: multiparas or primiparas. The “CC genotype”, “age<30” and “primipara” were treated as references for genotype, age group and parity, respectively.

Log-binomial regression was used to estimate the prevalence ratios (PR) using the logbin function (method = “em”) from the logbin package (v 2.0.5) in R 4.3.0, and the analysis of covariance (ANCOVA), using the ANOVA function (type = “III”) from the car package (v 3.1.2) in R 4.3.0, was applied to assess the association of *MTHFR* C677T genotype with the glucose levels due to its ability to control the confounding effects, which would result in a smaller variance than ANOVA (19).

#### 2.5.2 Interventional recovery analysis

Independent student’s t-tests were used when comparing the differences in means between the ages, blood glucose measurements, routine blood tests and hepatorenal function test results between the high-dose and the standard-dose group. The independent-samples t-test function in SPSS (v22) with default parameters.

The Kaplan-Meier estimator was commonly used for estimating the survival function. Here we applied this technique to investigate the probability of GDM persistence during the folic acid treatment period. Although a single negative OGTT result is considered sufficient to ensure that GDM has resolved, to achieve higher confidence, we also extracted the patients with at least two OGTT results and re-defined ‘recovery’ as two consecutive negative test results. Both the log-rank test and cox’s proportional hazards model were used to test if there is a difference in length of recovery between the high-dose group and the standard-dose group using the cox-regression function in SPSS (v22).

The rate ratio (RR) of GDM recovery was defined as the ratio between the recovery rates of the high-dose group and the standard-dose group.


$$RR=RR^{\left(t\right)}=\frac{R_{E}^{\left(t\right)}}{R_{C}^{\left(t\right)}}=\frac{X_{E}^{\left(t\right)}/N_{E}^{\left(t\right)}}{X_{C}^{\left(t\right)}/N_{C}^{\left(t\right)}}$$

where t stands for the period starting from when the first OGTT was conducted to the day of the last OGTT test before delivery and *t ≤* 100; $X_{E}^{\left(t\right)}$ is the number of patients recovered from GDM in the high-dose group within t; $N_{E}^{\left(t\right)}$ is the total number of patients in the high-dose group within t; $X_{C}^{\left(t\right)}$ is the number of patients recovered from GDM in the standard-dose group within t; $N_{C}^{\left(t\right)}$ is the total number of patients in the standard-dose group within t. For simplicity, we will omit the superscript t for any downstream equations.

The confidence interval of RR was calculated by first transforming to the log scale so that it can be estimated by the normal distribution (20):


$$\text{ln}\left(RR\right)\pm z_{1-\frac{\alpha }{2}}\left(SE_{lnRR}\right)$$

where


$$SE_{lnRR}=\sqrt{\frac{1}{X_{E}}-\frac{1}{N_{E}}+\frac{1}{X_{C}}-\frac{1}{N_{C}}}$$

Figures were made with either GraphPad Prism 9 or the ggplot2 package (v 3.3.5) in R 4.0.5.

## 3 Results

### 3.1 No significant association between *MTHFR* 677 genotype and GDM risk

**Figure 2:**
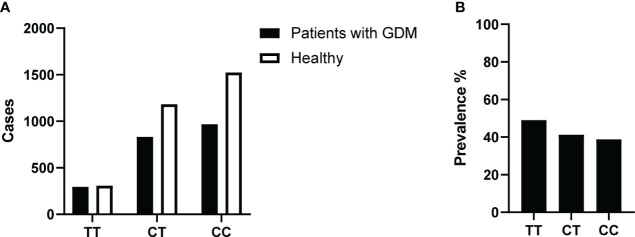
**(A)** Number of GDM and healthy cases in recruited study participants and their *MTHFR* 677 genotypes. Pregnant women were tested at 24 weeks of gestation with all three blood glucose results and reviews of certain abnormal values were included. **(B)** Prevalence of GDM at 24 weeks of pregnancy. % Prevalence of GDM in the three different *MTHFR* genotype groups: TT/CT/CC.

We first investigated the relationship between the *MTHFR* C677T gene polymorphism and the GDM risk. The analysis was done based on the 5,111 pregnancy events with both *MTHFR* 677 genotypes and the GDM screening results available (**Figure 2A**). We calculated the prevalence of GDM around 24 weeks of gestation for each of the *MTHFR* 677 TT/CC/CT genotype using a contingency table and found a higher prevalence of GDM in pregnant women with the homozygous TT genotype (49.01%) (**Figure 2B**). Logistic regression was conducted to estimate the odds ratios (ORs). With a null model, ORs of GDM between TT and CC and between CT and CC were 1.513 (95% CI 1.265, 1.809; p<0.001) and 1.110 (95% CI 0.984, 1.251); p=0.089), respectively. However, after adjusting for age and parity, the ORs of *MTHFR* C677T polymorphism were 1.105 (95% CI 0.853, 1.431; p=0.452) between TT and CC and 1.082 (95% CI 0.916, 1.278; p=0.352) between CT and CC. Hence, no significant association was found between the *MTHFR* phenotypes and the GDM risk (**Table 1**). In case logistic regression overestimates the ORs due to inadequate sample size or when the prevalence is greater than 10% (21), we further calculated the prevalence ratios (PRs) to have a better estimation of the GDM risk using the log binomial model. With high consistency, after adjusting for age and parity, *MTHFR* 677 TT and CT mutants acquired PRs close to 1 (TT: 1.050 (95% CI 0.906, 1.216; p=0.518) and CT 1.039 (95% CI 0.941, 1.147; p=0.447)) with the wildtype CC group as the reference. But instead, maternal age and parity could be important risk factors positively associated with the GDM risk, with a PR of 1.935 (95% CI 1.717, 2.181) (age>34 versus age<30), and 1.213 (95% CI 1.093, 1.346) (multipara versus primipara) (**Table 1**).

**Table 1:** Estimations of the risk of GDM between patients with different *MTHFR* 677 genotypes.

	Odds Ratio (Logistic)			Prevalence Ratio (Log-binomial)		
Covariate	OR	95% CI	p	PR	95% CI	p
Age Group (30-34)	1.848	1.554-2.199	<0.001	1.481	1.331-1.649	<0.001
Age Group (>34)	3.209	2.512-4.099	<0.001	1.935	1.717-2.181	<0.001
Multipara	1.393	1.149-1.688	0.001	1.213	1.093-1.346	<0.001
Genotype (CT)	1.082	0.916-1.278	0.352	1.039	0.941-1.147	0.447
Genotype (TT)	1.105	0.853-1.431	0.452	1.049	0.906-1.216	0.518

### 3.2 *MTHFR* 677 genotype is not significantly associated with the glucose levels in pregnant women

GDM was defined based on three glucose measurements, including FBG and PBG (at 1h and 2hr). Although the *MTHFR* 677 genotypes may not be directly associated with the GDM risk, the downstream effects at the glucose levels are still unclear. To have a better control of the confounding effects of maternal age and parity (22), an ANCOVA model was used with maternal age and parity as covariates, results of which suggested that *MTHFR* 677 genotypes are likely not a significant determinant of blood glucose control with a p-value of 0.225, 0.738 and 0.594, when assessing the FBG, PBG(1h) and PBG(2h) levels, respectively. Again, the maternal age was shown to be an important risk factor for the blood glucose levels with all p-values<0.001, and parity was associated with FBG (p<0.001) and PBG(2h) (p<0.05).

### 3.3 High folic acid supplement after 24 weeks of pregnancy reduced GDM risk and showed better GDM relief in patients with the *MTHFR* 677 TT genotype

Since *MTHFR* is an enzyme involved in the folic acid metabolic pathway and its 677 TT genotype results in a higher GDM risk, this raises the question: can GDM conditions be reversed with higher dose of folic acid supplements? To address this query, we designed a prospective interventional study with carefully selected participants: pregnant women diagnosed with GDM with the *MTHFR* 677 TT genotype at 24-28 weeks of pregnancy. With written consent, the participants were arranged into two groups: a high-dose group prescribed high-dose of folic acid supplements (800μg/day, n=38) and a standard-dose group maintained with standard dose of folic acid supplements (400μg/day, n=133). The two groups are fully comparable, confirmed by comparing their baseline characteristics, type of glucose measurements taken, distribution of participants over time (the time of clinical events), routine blood tests and hepatorenal function test results prior to the start of the study as a baseline and at the end of the study before delivery (**Table 2**, **Supplementary Figure 1**, **Supplementary Figure 2**, **Supplementary Table 1** and **Supplementary Table 2**).

**Table 2:** Summary of basic information of patients selected for the interventional study.

	High-dose (n=38)		Standard-dose (n=133)		T-test	
Value(n)	Avg	SD	Avg	SD	t	p
Age	29.50(18)	4.27	30.00(113)	5.26	-0.383	0.702
FBG (mmol/L)	5.10(38)	0.60	5.05(133)	0.44	0.407	0.043
1h PBG (mmol/L)	10.23(38)	1.62	9.26(133)	1.55	3.370	0.001
2h PBG (mmol/L)	8.94(38)	1.85	7.94(133)	1.62	3.326	0.001

T-test results are compatible with homogeneity tests of variance. FBG, fasting blood glucose. PBG, postprandial blood glucose.

**Figure 3:**
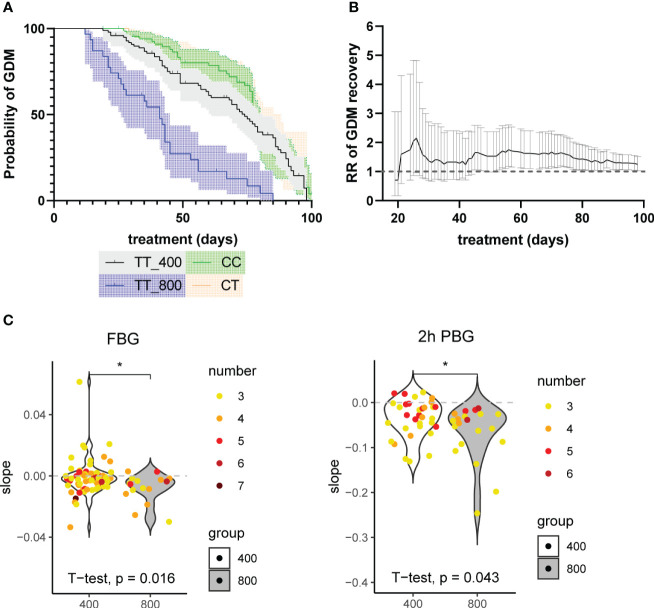
**(A)** Probability of freedom from GDM curves with Kaplan-Meier model. An *MTHFR* patient’s recovery from GDM (approach 2: the first two consecutive OGTT test results were both negative) was defined as an event. The little vertical bars on top of the lines represent deleted data, from patients who had not recovered from GDM in the last BG review results. The y-axis represents the estimated probability of GDM (unrecovered). The x-axis represents healing time, defined as the period between the event and the beginning of treatment. The shadow represents 95% CI. The log-rank test results were: TT_400 v TT_800: p<0.001; TT_400 v CC: p=0.317; TT_400 v CT: p=0.229; CT v CC: p=0.692. **(B)** The rate ratios (RRs) of GDM recovery of the high-dose group (800μg/day) compared with the standard-dose group (400μg/day) during treatment. The X-axis represents the folic acid supplementation treatment duration, calculated as the period between the last and the first glucose examination of every patient. Using data from patients with treatment time equal to or less than 100 days. RRs were calculated using all the examination data before each point in time as an indicator of the accumulative effect of the high-dose folic acid supplement. The range represents 95% CI. **(C)** The slopes of time-BG linear regression results of the patients. Patients with 3 or more fasting blood glucose (FBG) or 2 hours postprandial glucose (2h PBG) results (including the OGTT test results at inclusion) were included. The numbers of records used are plotted in different colors. Linear regression models were fitted with treatment time (days) as the x-axis and blood glucose (mmol) as the y-axis. *: p<0.05.

To compare the overall rates of GDM relief between the two groups, a Kaplan-Meier model was fitted. The Kaplan-Meier estimator is commonly used in survival analysis. Here instead, the same method was applied to estimate the probability of GDM persistence from the day of the first OGTT test to 100 days after. We used two approaches to define ‘GDM relief’: 1) if the first OGTT follow-on test result was negative. However, since results for OGTT may indicate GDM again after a period of temporary relief, to have higher confidence, alternatively, the second approach was conducted as 2) if the first two consecutive OGTT test results were both negative. The survival curves from both approaches agreed well. They showed that patients with higher doses of folic acid supplement recovered from GDM faster than those in the standard-dose group (**Figure 3A**, **Supplementary Figure 3**). The difference in recovery curves is more striking using Approach 2 (**Figure 3A**) with an average recovery time of 40.69 days (95% CI 33.22, 48.15) for the high-dose group compared with 68.09 days (95% CI 63.08, 73.11) for the standard-dose group with p<0.001 estimated using a log-rank test. The data of GDM patients with *MTHFR* 677 CC or CT genotypes were used as additional negative controls under the assumption that these patients also took standard doses of folic acid supplement (400μg/day) throughout their pregnancies. Although patients recovered from GDM gradually in the third trimester of pregnancy, the *MTHFR* TT genotype is likely more susceptible to folic acid supplements than CT/CC groups. However, p values estimated from log-rank tests were not significant potentially due to high variance (TT_400 v CT: p=0.229 and TT_400 v CC: p=0.317). To further investigate the differences between the recovery rates, cox regression was used to calculate the recovery ratio, which was 3.679 (95% CI 2.310, 5.857; p<0.001), with the standard-dose group as reference. All the above results further confirmed what we observed from the recovery curves patients supplied with higher doses of folic acid supplements recovered faster.

To further investigate if the length of folic acid treatment has an impact on the rates of GDM recovery between the two groups, we calculated RRs of GDM recovery at each available time point. The last glucose test result before delivery was considered as the final outcome. Our time series data revealed that correlating with the treatment, RR rose and reached 1.247 (95% CI 1.026, 1.515) at 100 days of treatment with the standard-dose group as reference. Patients on higher doses of folic acid intake show higher RRs of GDM recovery, especially within the first 30 days of treatment (**Figure 3B**). Since this is an overall trend for all participants and only the last glucose test record of each participant was used as the final outcome, to account for the interpersonal variability, we extracted the participants that have at least three glucose test records and estimated the trends that show the changes of glucose levels over time using linear models for each participant. The gradients were plotted and differences between means of high-dose and standard-dose folic acid intake for FBG and 2h PBG were compared using independent two sample t-tests. The results revealed lower levels of the FBG (p=0.016) and 2h PBG (p=0.043) in the high-dose group compared to the standard-dose group (**Figure 3C**).

## 4 Discussion and conclusion

From our data collected from a hospital in China for two years from 2017-2019, 41% of pregnant women who underwent OGTT were diagnosed with GDM, among which 14.12% are associated with an *MTHFR* C677 homozygous mutation. This incidence rate of GDM is higher than any reported statistics. It sends a warning message to the public and the healthcare system that GDM has emerged as a persistent problem during pregnancy. Insulin therapy remains the standard of care for uncontrolled GDM during pregnancy and commonly requires needle injection of insulin, which brings several disadvantages, such as the risk of hypoglycemia, inconvenience of use, strict storage requirements, and trypanophobia. Thus, alternative strategies are urgently in need for the management of GDM during pregnancy, especially in patients with the *MTHFR* 677 TT genotype that was previously reported as an important risk factor for GDM (16).

Using the data from 5,111 pregnancies diagnosed with GDM at 24-28 weeks, no association was found between the *MTHFR* 677 genotype and the GDM risk, taking into consideration age and parity as covariates in the model. On the other hand, the relationship between folic acid supplement intake before or during pregnancy and the development of GDM is still under debate. The standard recommended dose of folic acid supplement per day is 400μg/day during preconception and pregnancy. Several studies have reported that high folic acid supplement intake (>400μg/day) before or during pregnancy is associated with an increased risk of developing GDM in the second-third trimester than taking non/low/inadequate folic acid supplementation (<400μg/day) (23). In a more recent prospective cohort study, Li et al. provided an opposing viewpoint with further adjustment for other major dietary factors, such that each 100μg/day increase in supplemental folate intake results in a relative risk of 0.95 (0.92, 0.98) for GDM based on supplemental folate ranging from 0 to 1000μg/day (6). Furthermore, they claimed that food folate intake was not associated with GDM risk. Nevertheless, all the above suggest an association between folic acid supplement intake and the risk of developing GDM and this is unlikely to be associated with food folate intake. Therefore, we proposed a prospective case-control intervention study from a new aspect. Instead of looking into the risk of developing GDM, we recruited participants diagnosed with GDM and *MTHFR* 677 TT mutation in their second-third trimester, and compared high (800μg/day) vs standard (400μg/day) folic acid supplement intake in association with the GDM relief. The results showed a faster recovery rate with high-dose folic acid intake, especially within the first 30 days. Cumulative analysis of prevalence ratios at each time point confirmed an initial increase in GDM recovery. The trend stays consistent from 30 days onwards with a positive ratio of 1.247 (95% CI 1.026, 1.515) at 100 days of treatment using the standard group. Thus, as an initial conclusion, high dose folic acid supplement intake since mid-late pregnancy of women with *MTHFR* 677 TT genotype is significantly associated with faster recovery from GDM. A quick assessment of glucose changes over the treatment period for each participant using linear models also revealed the same pattern that FGB and PBG (2h) levels are lower in GDM patients taking high-dose of folic acid supplements.

The two groups in the study were fully comparable with matched age and inclusive/exclusive criteria. We excluded interfering factors by selecting participants restricted to GDM and hypoglycemia diagnoses, who had not been taking any prescribed medications apart from the folic acid supplements or multi-vitamins and did not have other chronic diseases. In addition, according to our records, no side or adverse effects were observed from either 400µg or 800µg folic acid intake through the intervention, and all pregnancies in this study were under satisfactory conditions and no adverse effect on the fetus from different doses of folic acid intake. However, long-term observational studies are needed to assess whether there are any adverse impacts of the high-dose folic acid supplement on newborns. Folic acid can be easily acquired from local pharmacies as multi-vitamin tablets (24) or prescribed as a drug (25), both of which should be cost-effective (~0.2 US dollars/1mg), especially for women from low- and middle-income countries (LMICs).

However, there are several inevitable limitations that need to be considered. Firstly, our sample size, especially for the high-dose group, was limited as a prospective cohort. It would be better recommended to increase the sample size and further include a high-dose group in GDM patients with *MTHFR* 677 CC or CT genotype as more precise negative controls. Nevertheless, this is a prospective and tentative clinical trial involving folic acid intervention and hence, would provide a trend that can be used as a guidance for the design of further large cohort studies. Secondly, when and how often OGTT were conducted are patient-specific. To overcome this, only patients with more than two OGTT test results were selected, with one between 24-28 weeks of pregnancy and one after 24-28 weeks but before delivery. If multiple OGTT were done, we were only restricted to the last test result as the final outcome for the analysis. Thirdly, this is a single center study with a geographical limitation that was restricted to China. Thus, although the findings could be applicable, a more diverse study would need to be conducted. Moreover, although Li et al. stated that food folate is not associated with the risk of GDM, the confounding of dietary folate intake is still a potential influence factor. According to a recent study conducted in China, the average dietary folic acid intake of women in early pregnancy is 145.4μg/day (P25, P75: 101.9, 200.7) (26), while Yu et al. revealed that individualized folic acid supplementation based on the polymorphisms of *MTHFR* may be a powerful measure to reduce GDM (27). Two cohort studies in northern Europe showed difference in association with GDM between self-reported and prescribed folic acid (28). We assumed that folic acid intake from food contributes to both groups homogenously. However, diet interventions could be considered during pregnancy to eliminate this influence in future experimental designs. Conversely, the untypical features of GDM have caused many obstacles in reaching significant statistical conclusions, including processing laboratory test results and measuring hazard factors. More efforts are yet to be made to progress the diagnosis and treatment of GDM. Finally, due to ethnical restrictions, the intervention was not randomly allocated or blinded, hence, the results and conclusions in this study would need to be treated with caution.

In summary, we found that high-dose (800μg/day) folic acid supplement intake in the second-third trimester during pregnancy is associated with better GDM relief in patients with GDM and the *MTHFR* 677 TT mutation compared to the standard dose (400μg/day). The results would provide guidance to future clinical trials that high-dose folic acid supplementation in mid-late pregnancy could offer pregnant women with TT genotype an improved and low-cost avenue for faster GDM relief.

## Data availability statement

The datasets presented in this article are not readily available because of privacy restrictions. Requests to access the datasets should be directed to Jun Ying, sjtuyj1636@sjtu.edu.cn.

## Ethics statement

The studies involving humans were approved by the Ethics Committee of the University of Chinese Academy of Sciences, Shenzhen Hospital. The studies were conducted in accordance with the local legislation and institutional requirements. The participants provided their written informed consent to participate in this study.

## Author contributions

JY, JZ and HZ: Conceptualization, formal analysis, methodology, writing-original draft. PL, LL, YL, WL, BW and QC: Investigation (diagnosis, inclusion, follow-up), data collection and curation, project administration. XW and HZ: Conceptualization, methodology, supervision, writing-original draft. All authors contributed to the article and approved the submitted version.
