# Supplementary material for: Enhanced recovery in patients with gestational diabetes mellitus and MTHFR 677 TT genotype after taking high-dose folic acid supplements during mid-late pregnancy: an open-label interventional study
Source: Front Endocrinol (Lausanne). 2023 Sep 25;14:1007192. doi: 10.3389/fendo.2023.1007192 (PMC10561647; doi:10.3389/fendo.2023.1007192)
Supplement: Supplementary file 1 [file DataSheet_1.docx]

Supplementary Material

Supplementary Table 1—The latest blood, liver and kidney function test results of the patients before inclusion

A. Blood routine test

|  | High-dose(n=38) | | Standard-dose(n=133) | | T-test | |
| --- | --- | --- | --- | --- | --- | --- |
|  | Avg(n=17) | SD | Avg(n=57) | SD | t | p |
| BASO# (10^9^/L) | 0.02 | 0.01 | 0.02 | 0.01 | -0.205 | 0.838 |
| BASO% | 0.25 | 0.09 | 0.25 | 0.11 | 0.051 | 0.960 |
| EO# (10^9^/L) | 0.14 | 0.12 | 0.14 | 0.12 | 0.084 | 0.933 |
| EOS % | 1.46 | 1.31 | 1.38 | 1.39 | 0.226 | 0.822 |
| HCT (%) | 34.16 | 3.30 | 35.47 | 2.72 | -1.651 | 0.103 |
| HGB (g/L) | 115.76 | 12.52 | 118.58 | 9.91 | -0.966 | 0.337 |
| LYM# (10^9^/L) | 7.67 | 2.18 | 7.72 | 2.61 | -0.068 | 0.946 |
| LYM% | 20.62 | 5.45 | 19.46 | 4.98 | 0.825 | 0.412 |
| MCH (Pg) | 28.49 | 3.60 | 29.97 | 2.51 | -1.909 | 0.060 |
| MCHC (g/L) | 338.47 | 14.20 | 334.42 | 10.38 | 1.292 | 0.200 |
| MCV (fL) | 84.01 | 8.75 | 89.57 | 6.72 | -2.782 | 0.007 |
| MONO# (10^9^/L) | 0.48 | 0.17 | 0.52 | 0.18 | -0.885 | 0.379 |
| MONO% | 4.63 | 1.09 | 5.12 | 1.29 | -1.437 | 0.155 |
| MPV (fL) | 9.22 | 1.12 | 9.41 | 1.36 | -0.517 | 0.606 |
| NEU# (10^9^/L) | 7.67 | 2.18 | 7.72 | 2.61 | -0.068 | 0.946 |
| NEU% | 71.68 | 8.21 | 73.78 | 5.84 | -1.199 | 0.235 |
| PCT (%) | 0.20 | 0.04 | 0.20 | 0.04 | -0.020 | 0.984 |
| PDW (fL) | 15.92 | 0.37 | 15.56 | 2.08 | 0.712 | 0.479 |
| PLCR (%) | 21.42 | 7.67 | 22.51 | 9.48 | -0.434 | 0.666 |
| PLT (10^9^/L) | 218.12 | 47.95 | 215.00 | 50.19 | 0.227 | 0.821 |
| RBC (10^12^/L) | 4.12 | 0.49 | 3.99 | 0.46 | 1.086 | 0.281 |
| RDWCV (%) | 13.45 | 1.39 | 13.47 | 1.49 | -0.057 | 0.955 |
| RDWSD (fL) | 40.64 | 4.56 | 42.99 | 4.21 | -1.985 | 0.051 |
| WBC (10^9^/L) | 10.33 | 2.56 | 10.31 | 2.92 | 0.026 | 0.979 |

B. Liver and kidney function test

|  | High-dose(n=38) | | Standard-dose(n=133) | | T-test | |
| --- | --- | --- | --- | --- | --- | --- |
| Value(n) | Avg | SD | Avg | SD | t | p |
| ALB (g/L) | 38.43(16) | 2.89 | 39.31(42) | 3.19 | -0.970 | 0.336 |
| ALT (U/L) | 17.00(17) | 13.67 | 16.73(45) | 12.22 | 0.074 | 0.941 |
| AST (U/L) | 18.29(7) | 3.40 | 19.36(25) | 8.99 | -0.307 | 0.761 |
| DBIL (μmol/L) | 1.87(11) | 1.40 | 2.21(21) | 0.95 | -0.807 | 0.426 |
| GGT (U/L) | 11.5(10) | 5.13 | 14.32(19) | 8.20 | -0.985 | 0.334 |
| TBA (μmol/L) | 1.49(15) | 0.76 | 2.41(41) | 2.00 | -1.739 | 0.088 |
| TBIL (μmol/L) | 9.86(16) | 4.60 | 9.84(42) | 3.41 | 0.022 | 0.982 |
| TP (g/L) | 65.14(16) | 4.19 | 66.47(42) | 4.51 | -1.028 | 0.308 |
| BUN (mmol/L) | 2.52(16) | 0.64 | 2.57(42) | 0.66 | -0.249 | 0.804 |
| Cr (μmol/L) | 40.42(17) | 5.53 | 42.26(45) | 8.50 | -0.824 | 0.413 |
| UA (μmol/L) | 231.05(17) | 55.44 | 228.41(45) | 69.24 | 0.141 | 0.888 |

Supplementary Table 2—The last blood, liver and kidney test results of the patients in the experiment

A. Blood routine test

|  | High-dose(n=38) | | Standard-dose(n=133) | | T-test | |
| --- | --- | --- | --- | --- | --- | --- |
|  | Avg(n=14) | SD | Avg(n=24) | SD | t | p |
| BASO# (10^9^/L) | 0.03 | 0.01 | 0.02 | 0.01 | 0.827 | 0.413 |
| BASO% | 0.28 | 0.11 | 0.29 | 0.22 | -0.205 | 0.839 |
| EO# (10^9^/L) | 0.26 | 0.33 | 0.14 | 0.10 | 1.645 | 0.109 |
| EOS% | 2.94 | 3.76 | 1.68 | 1.42 | 1.478 | 0.148 |
| HCT (%) | 36.77 | 4.05 | 37.13 | 3.36 | -0.290 | 0.774 |
| HGB (g/L) | 119.36 | 13.65 | 121.13 | 12.01 | -0.416 | 0.680 |
| LYM# (10^9^/L) | 5.91 | 1.68 | 6.10 | 2.10 | -0.297 | 0.769 |
| LYM% | 26.21 | 9.30 | 22.86 | 8.09 | 1.166 | 0.251 |
| MCH (Pg) | 27.32 | 3.81 | 29.18 | 2.81 | -1.720 | 0.094 |
| MCHC (g/L) | 324.71 | 12.37 | 326.21 | 7.29 | -0.412 | 0.685 |
| MCV (fL) | 84.04 | 10.44 | 89.39 | 7.51 | -1.834 | 0.075 |
| MONO# (10^9^/L) | 0.54 | 0.17 | 0.55 | 0.20 | -0.134 | 0.894 |
| MONO% | 6.05 | 1.67 | 6.32 | 1.71 | -0.476 | 0.637 |
| MPV (fL) | 9.96 | 1.49 | 9.32 | 0.94 | 1.459 | 0.161 |
| NEU# (10^9^/L) | 5.91 | 1.68 | 6.10 | 2.10 | -0.297 | 0.769 |
| NEU% | 64.52 | 9.78 | 68.85 | 8.99 | -1.385 | 0.175 |
| PCT (%) | 0.20 | 0.04 | 0.19 | 0.04 | 0.817 | 0.419 |
| PDW (fL) | 15.05 | 2.64 | 16.05 | 1.16 | -1.628 | 0.112 |
| PLCR (%) | 27.06 | 11.50 | 22.60 | 6.06 | 1.346 | 0.196 |
| PLT (10^9^/L) | 209.07 | 53.07 | 206.83 | 43.97 | 0.140 | 0.889 |
| RBC (10^12^/L) | 4.43 | 0.65 | 4.18 | 0.48 | 1.376 | 0.177 |
| RDWCV (%) | 13.89 | 1.03 | 13.96 | 1.80 | -0.145 | 0.885 |
| RDWSD (fL) | 41.25 | 4.50 | 43.93 | 5.01 | -1.651 | 0.107 |
| WBC (10^9^/L) | 9.05 | 1.76 | 8.67 | 2.16 | 0.553 | 0.583 |

B. Liver and kidney function test

|  | High-dose(n=38) | | Standard-dose(n=133) | | T-test | |
| --- | --- | --- | --- | --- | --- | --- |
| Value(n) | Avg | SD | Avg | SD | t | p |
| ALB (g/L) | 36.78(12) | 2.58 | 36.34(18) | 2.05 | 0.509 | 0.615 |
| ALT (U/L) | 10.5(12) | 5.71 | 9.67(18) | 3.07 | 0.519 | 0.608 |
| AST (U/L) | 17.6(5) | 5.18 | 15.58(12) | 3.45 | 0.951 | 0.357 |
| DBIL (μmol/L) | 1.70(7) | 0.61 | 1.60(6) | 0.81 | 0.253 | 0.805 |
| GGT (U/L) | 10.43(7) | 4.43 | 11.00(6) | 4.69 | -0.226 | 0.826 |
| TBA (μmol/L) | 2.74(12) | 2.08 | 3.47(18) | 3.45 | -0.656 | 0.517 |
| TBIL (μmol/L) | 10.98(12) | 3.61 | 13.39(18) | 5.10 | -1.415 | 0.168 |
| TP (g/L) | 63.94(12) | 5.14 | 63.55(18) | 3.57 | 0.247 | 0.807 |
| BUN (mmol/L) | 3.23(12) | 1.35 | 2.76(18) | 0.65 | 1.129 | 0.277 |
| Cr (μmol/L) | 45.46(12) | 9.79 | 46.28(18) | 5.43 | -0.295 | 0.770 |
| UA (μmol/L) | 312.71(12) | 81.12 | 286.06(18) | 57.88 | 1.052 | 0.302 |


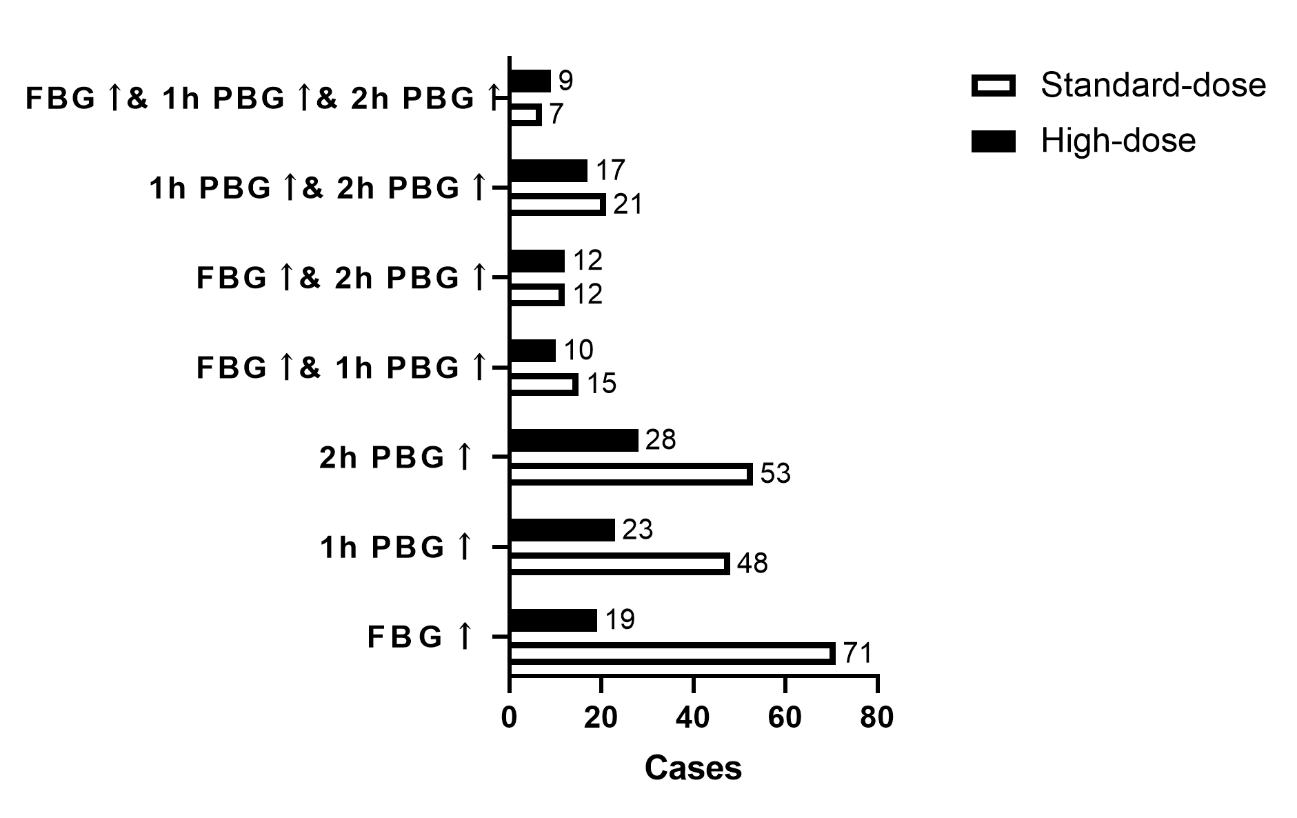


Supplementary Figure 1. The number of patients with abnormal blood glucose test results at inclusion. ↑represents fasting blood glucose (FBG) >5.1mmol/L or 1 hour postprandial blood glucose (1h PBG) >10.0mmol/L or 2 hours postprandial blood glucose (2h PBG) >8.5mmol/L, respectively.


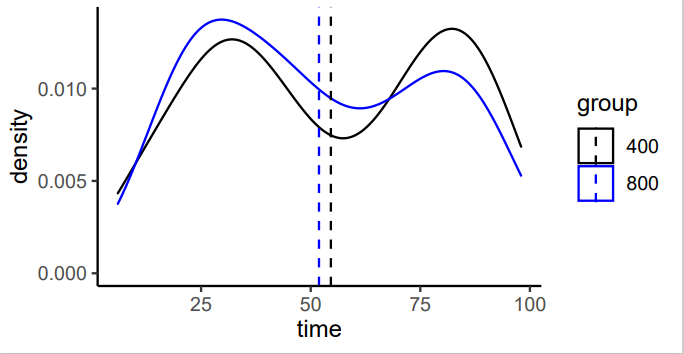


Supplementary Figure 2. The density distribution of recovered and maintained GDM cases during treatment (For Fig. 6). X-axis represents treatment duration (days). Dashed lines represent means.


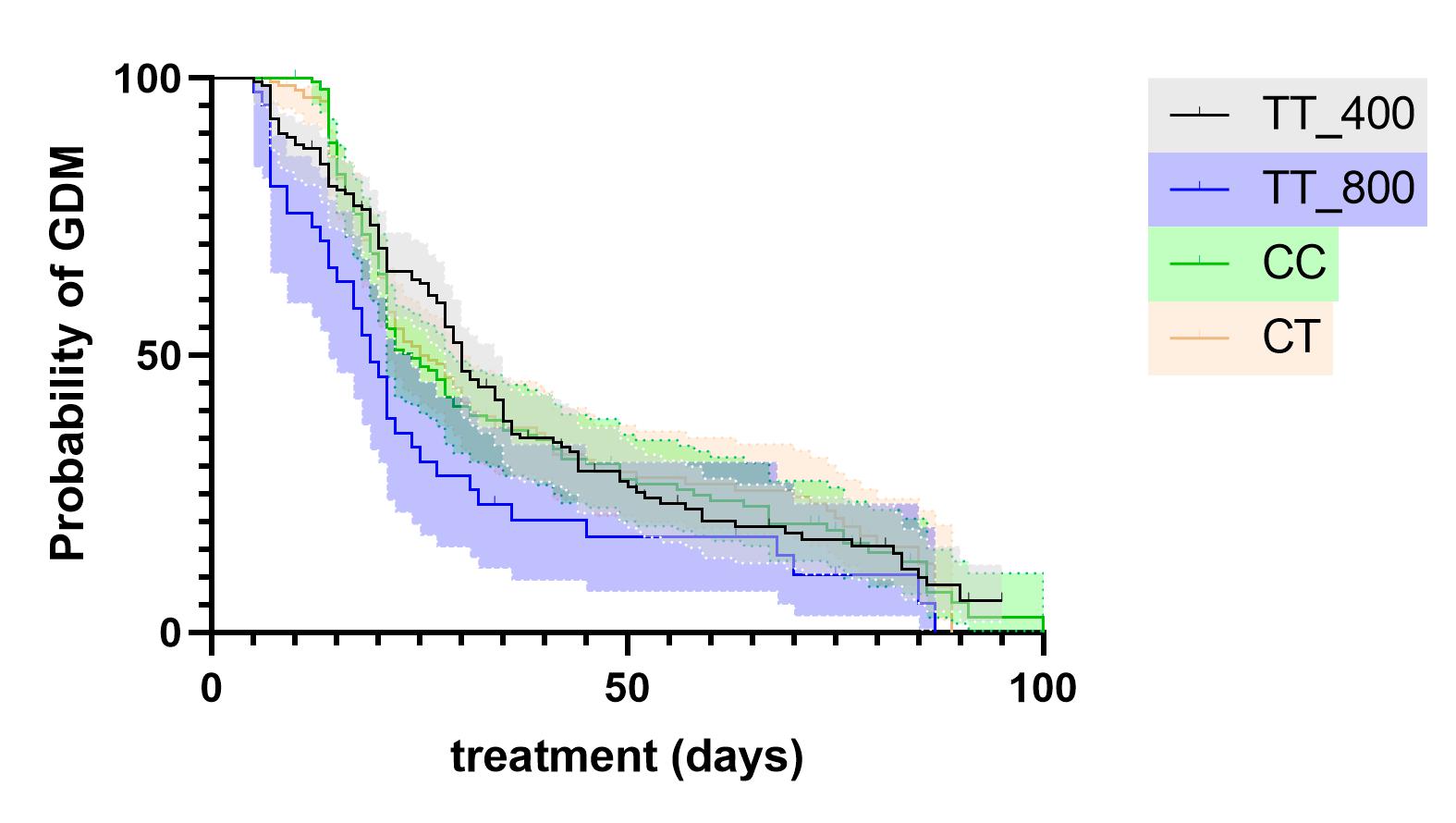


Supplementary Figure 3. Survival curves with Kaplan-Meier model. The same as Fig. 5, but using approach 1 (the first OGTT follow-on test result was negative) instead. The log-rank test results were: TT_400 v TT_800: p=0.011; TT_400 v CC: p=0.709; TT_400 v CT: p=0.842; CT v CC: p=0.841.
